# A simulation study on estimating biomarker–treatment interaction effects in randomized trials with prognostic variables

**DOI:** 10.1186/s13063-018-2491-0

**Published:** 2018-02-20

**Authors:** Bernhard Haller, Kurt Ulm

**Affiliations:** Institute of Medical Informatics, Statistics and Epidemiology, Technical University of Munich, Ismaninger Str. 22, Munich, 81675 Germany

**Keywords:** Biomarker–treatment interaction, Randomized trial, Stratified medicine, Predictive covariates, Variable selection

## Abstract

**Background:**

To individualize treatment decisions based on patient characteristics, identification of an interaction between a biomarker and treatment is necessary. Often such potential interactions are analysed using data from randomized clinical trials intended for comparison of two treatments. Tests of interactions are often lacking statistical power and we investigated if and how a consideration of further prognostic variables can improve power and decrease the bias of estimated biomarker–treatment interactions in randomized clinical trials with time-to-event outcomes.

**Methods:**

A simulation study was performed to assess how prognostic factors affect the estimate of the biomarker–treatment interaction for a time-to-event outcome, when different approaches, like ignoring other prognostic factors, including all available covariates or using variable selection strategies, are applied. Different scenarios regarding the proportion of censored observations, the correlation structure between the covariate of interest and further potential prognostic variables, and the strength of the interaction were considered.

**Results:**

The simulation study revealed that in a regression model for estimating a biomarker–treatment interaction, the probability of detecting a biomarker–treatment interaction can be increased by including prognostic variables that are associated with the outcome, and that the interaction estimate is biased when relevant prognostic variables are not considered. However, the probability of a false-positive finding increases if too many potential predictors are included or if variable selection is performed inadequately.

**Conclusions:**

We recommend undertaking an adequate literature search before data analysis to derive information about potential prognostic variables and to gain power for detecting true interaction effects and pre-specifying analyses to avoid selective reporting and increased false-positive rates.

**Electronic supplementary material:**

The online version of this article (doi:10.1186/s13063-018-2491-0) contains supplementary material, which is available to authorized users.

## Background

Treatment individualization, i.e. finding the right treatment with the right dose at the right time for a specific patient based on certain patient characteristics, is one of the great goals in modern medicine [1]. One requirement for treatment individualization based on, e.g. a certain biomarker like a genetic characteristic or a blood parameter, is the existence of a relevant association between the biomarker and the treatment effect [2], often referred to as the biomarker–treatment interaction. Only a small number of trials have been planned to analyse biomarker–treatment interactions [3], but often the association between one or more biomarkers and a treatment effect is evaluated post hoc in data collected in randomized clinical trials intended for overall comparison of treatment groups, like e.g. the detection of the association between the response to cetuximab and the presence or absence of the K-ras mutation in the tumours of patients with advanced colorectal cancer [4].

While often the treatment effect is analysed in different subgroups (pre-specified or post hoc specified) to identify patients that benefit from one or another treatment [5], it is widely recognized that the comparison of treatment groups in many different subgroups can lead to spurious results [6]. Therefore, it is often recommended to assess the biomarker–treatment interaction in a regression model, which directly allows us to estimate and test for an interaction effect under common model assumptions [7]. Various authors who provide methods for estimating biomarker–treatment interactions stress the importance of the adequate inclusion of prognostic factors in the model [8, 9]. For treatment effect estimation in a randomized clinical trial, the European Medicines Agency’s guideline on ‘Points to consider on adjustment for baseline covariates’ recommends including other prognostic factors, i.e. covariates that are assumed to be associated with the outcome, as covariates in the regression model to increase the precision of the estimate of the treatment effect [10]. Furthermore, it has been shown that the estimate for the treatment effect is biased in a Cox regression model, if relevant prognostic covariates are not included [11]. While defining the model used for effect estimation and hypothesis testing a priori and including all relevant covariates can be considered as best practices [12], adequate information about prognostic factors might not be available for all research questions, especially when molecular information that has not been well studied and for which limited information from prior investigations is available is included in a regression model. Various approaches to determining the covariates that are to be included in a regression model are presented in the literature [13].

The focus of this article is estimating the interaction between one certain pre-specified biomarker of major interest and the treatment. A simulation study was performed to evaluate how the presence and inclusion of further prognostic covariates affect the estimate of the biomarker–treatment interaction. Different strategies for model building, such as including only the main effects of treatment, the biomarker and their interaction, additionally including covariates that are significantly associated with the outcome, or using variable selection methods based on Akaike’s information criterion (AIC) [14] are considered. Scenarios with varying proportions of censored observations, different strengths of association of the prognostic covariates and the outcome, different correlations between prognostic covariates and the biomarker of interest, and different numbers of potential prognostic covariates are considered. The different strategies of covariate inclusion are compared in the control of type I error probabilities and the power to reject the null hypothesis of no biomarker–treatment interaction. A special focus was placed on the so-called rule of ten [12, 15]. This is often considered for predictive models, but (to the best of our knowledge) has not been investigated for the number of additional covariates considered in a regression model, when the primary goal was estimation of an interaction effect.

## Methods

### Assessing the biomarker–treatment interaction

The interaction between a continuous biomarker of major interest *B*, or a continuous covariate in general, and treatment *T*, which is assumed to be binary throughout the article (*T*∈{0;1}), can be assessed by including an interaction term between the biomarker and the treatment in an adequate regression model. This means the product of *B* and *T* is included in the regression model as an additional covariate (see e.g. [13]). The Cox regression model [16], also known as the proportional hazards model, is commonly considered in the analysis of survival data in medical research. In the Cox model, the effect of the biomarker *B*, the treatment *T*, their interaction *T*×*B* and *K* other covariates described through the matrix ***X***_***k***_ on the hazard rate *λ*(*t*) is modelled as

1 $$ \begin{aligned} \lambda(t|T\!, B, \boldsymbol{X_{k}}) \,=\, \lambda_{0}(t)\!\exp(\beta_{T}\!T\! +\! \beta_{B}B \,+\, \beta_{T\times B}T\times B \,+\, \boldsymbol{\beta_{k}^{T}} \boldsymbol{X_{k}}), \end{aligned}  $$

where a linear association between a covariate and the log-hazard ratio is assumed. In Eq. (1), *λ*_0_(*t*) is the (unspecified) baseline hazard rate, *β*_*T*_ the regression coefficient for treatment *T*, *β*_*B*_ the coefficient for the biomarker of interest *B*, *β*_*T*×*B*_ the regression coefficient for their interaction term and ***β***_***k***_ the vector of regression coefficients for the *K* additional covariates, *X*_1_,…,*X*_*K*_. When an interaction term is present, the main effects of the treatment *T* and the biomarker *B* can be interpreted as the expected treatment difference at a (fictitious) biomarker value of *B*=0 and the effect of the biomarker *B* under treatment *T*=0 conditional on all other covariates. Regression coefficients are estimated by numerical maximization of the partial log-likelihood PL(***β***). The variance-covariance matrix of the estimated regression coefficients can be derived as the inverse of the observed information matrix $ {I}^{-1}(\boldsymbol {\hat {\beta }})$ (see e.g. [16] or [17] for more details).

### Strategies for covariate inclusion

In the simulation study, various approaches for including covariates are compared. In all models, the main effects of the treatment and biomarker as well as their interaction term are included. Obviously, the best choice would be to fit the true model to the data, which includes all covariates that are truly associated with the outcome and ignoring those covariates that are not. This model will be estimated using the simulated data, but in practice the true model will not be known and therefore, the model must be chosen based on plausibility and previous knowledge or based on information gathered from the observed data. Therefore, the following models and strategies were investigated. The names are used for the models/strategies in the figures and tables presented in this article:

- Main: A model including only the main effects of treatment *T* and the biomarker *B* and their interaction *T*×*B*, ignoring all other possible prognostic covariates.
- True: A model including the main effects of treatment *T*, the biomarker of interest *B* and their interaction *T*×*B*, as well as all covariates that are truly associated with the outcome, indicating perfect prior knowledge of relevant covariates.
- AIC _*A*_: A model that includes the main effects of treatment *T* and the biomarker *B* and their interaction *T*×*B* and additionally all covariates that were selected in a forward variable selection procedure based on Akaike’s information criterion (AIC) [14] given *T*, *B* and *T*×*B* are included (a model including *T*, *B* and *T*×*B* was used as a starting and minimal model). Additional covariates were selected as long as the AIC criterion
  2 $$ \operatorname{AIC} = 2\operatorname{ll}(\hat{\boldsymbol{\beta}}) - 2p  $$
  was increased, where $\operatorname {ll}(\hat {\boldsymbol {\beta }})$ is the partial log-likelihood evaluated at the maximum likelihood estimator $\hat {\boldsymbol {\beta }}$ and *p* is the number of estimated regression coefficients.
- AIC _*B*_: A modelling strategy similar to AIC _*A*_ described above, but prognostic factors were selected based on the AIC criterion considering just the main effect of treatment *T* as a starting model and not including *B* or *T*×*B* in the variable selection process. After prognostic factors were chosen according to the AIC criterion, *B* and *T*×*B* were added to the model to estimate the biomarker–treatment interaction.
- Significance: A model that includes the main effects of treatment *T*, the covariate of interest and their interaction, as well as all covariates that were significantly associated with the outcome in a Cox regression model including only one covariate (often referred to as univariate Cox models in the medical literature). While this strategy is generally not recommended from a statistical point of view [18], it appears to be a quite popular approach in practice.
- Full: A model that includes the treatment *T*, the biomarker *B* and their interaction *T*×*B* as covariates as well as the main effects of all *K* potential predictors *X*_1_,…,*X*_*K*_.

### Data generation and simulation settings

Numerous different settings were considered to evaluate the modelling strategies under varying conditions. For each simulation scenario, 500 subjects were generated. The matrix of continuous covariates (covariate of interest *B* and potential predictors *X*_1_,…,*X*_*K*_) was drawn from a multivariate normal distribution using the R package *mvtnorm* [19]. For each variable, a mean of 0 and a standard deviation of 1 were used. The correlation structure was specified as described below. Since a randomized controlled trial was intended to be simulated, the treatment variable was drawn independently from all other patient characteristics with Pr(*T*=1)= Pr(*T*=0)=0.50 for each individual. For all scenarios, *β*_*T*_ and *β*_*B*_ were chosen as *β*_*T*_= ln(0.75)=−0.288 (i.e. exp(*β*_*T*_)=0.75) and *β*_*B*_= ln(1.25)=0.223 (i.e. exp(*β*_*B*_)=1.25).

For each scenario, a time-constant baseline hazard rate of *λ*_0_(*t*)=1 was used. The hazard rate for each individual was calculated according to Eq. 1 considering the patient’s characteristics and the regression coefficients for the specific scenario. Event times were generated from an exponential distribution using each individual’s hazard rate. All aspects of the simulation study including data generation, estimating regression coefficients and summarizing the results were performed with the statistical software R [20].

The following aspects were varied in the simulation study.

**Censoring distribution**

Administrative censoring after 5 years was assumed for all scenarios. Additionally, censoring times were generated independently of the event times from an exponential distribution. The hazard rate of the censoring distribution was chosen to produce scenarios with

1. a low proportion of censored observations (between 30% and 40% censored observations corresponding to 300 to 350 observed events)
2. a high proportion of censoring (between 60% and 70% censored observations corresponding to 150 to 200 observed events).

**Strength of interaction**

**Fig. 1:**
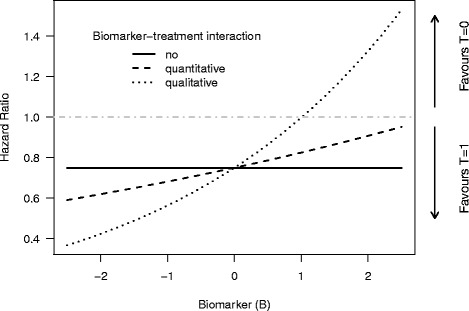
Illustration of the different strengths of interaction used in the simulation study. A hazard ratio larger than 1 indicates a higher risk for death under treatment *T*=1, and a hazard ratio below 1 a higher risk under treatment *T*=0. For the scenario with no biomarker–treatment interaction, the hazard ratio between the treatment groups is independent of the biomarker value. For the scenario with a quantitative biomarker–treatment interaction, the risk for an event is smaller under *T*=1 compared to *T*=0 for all (probable) values of *B*, but the difference between groups decreases with increasing values of *B*. For the scenario with a qualitative biomarker–treatment interaction, the risk for an event is lower for *T*=1 compared to *T*=0 for small values of *B* and vice versa for large values of *B*

The strength of the interaction effect between the covariate of interest *B* and treatment *T* was varied to consider scenarios with no, quantitative or qualitative biomarker–treatment interaction [21] (see also Fig. 1):

1. Simulation of data under the null hypothesis of no biomarker–treatment interaction: *β*_*T*×*B*_=0.
2. Quantitative biomarker–treatment interaction with a difference in the magnitude of the treatment effect between individuals with a low value of *B* and individuals with a large value of *B*: *β*_*T*×*B*_= ln(1.1)=0.095, leading to a hazard ratio between the treatment groups (*T*=1 vs. *T*=0) of about 0.6 for a given value of *B*=−2 and a hazard ratio of about 0.9 for *B*=2.
3. Qualitative biomarker–treatment interaction indicating an expected lower risk for an event from treatment *T*=1 for patients with a small value of *B* and a lower risk under treatment *T*=0 for patients with a large value of *B*: *β*_*T*×*B*_= ln(1.33)=0.285, providing a hazard ratio between the treatment groups smaller than 1 for *B*<1 and a hazard ratio larger than 1 for *B*>1 (dotted line in Fig. 1).

**Number of potential prognostic variables to be included in the model** Three settings for the number *K* of potential candidate predictors that can be included in the regression model were considered:

1. *K*=12: Here 12 additional prognostic covariates are considered, so the rule of ten is fulfilled under both censoring distributions for most simulation runs, as 150 to 200 events are expected in the settings with a high amount of censoring and up to 15 regression coefficients are to be estimated (12 prognostic variables plus the main effects of treatment *T* and the covariate of interest *B* and their interaction *T*×*B*).
2. *K*=24: Here 24 additional prognostic covariates are considered, so the rule of ten will be violated for most scenarios with high censoring.
3. *K*=36: Here 36 additional prognostic covariates are considered. Again, the rule of ten will be violated under high censoring.

**Correlation structure between prognostic variables and covariate of interest** Three different correlation structures between the covariate of interest *B* and the potential prognostic variables *X*_1_,…,*X*_*K*_ were considered:

1. Firstly, a scenario with a biomarker of interest *B* that is independent of the potential prognostic variables, and independence between all the prognostic variables was investigated, with
  $$\Sigma_{1} = \left(\begin{array}{ccccc} 1 & 0 & \cdots & \cdots & 0 \\ 0 & 1 & 0 & \cdots & 0 \\ \vdots & & \ddots & &\vdots \\ 0 & \cdots & 0 & 1 & 0 \\ 0 & \cdots & \cdots & 0 & 1 \\ \end{array} \right). $$
2. As a second setting, the correlation coefficients between *B* and all other covariates *X*_1_,…,*X*_*K*_, as well as between each pair of covariates *X*_*i*_,*X*_*j*_ with *i*≠*j* was set to *r*=0.5, indicating a moderate correlation between all variables:
  $$\Sigma_{2} = \left(\begin{array}{ccccc} 1 & 0.5 & \cdots & \cdots & 0.5 \\ 0.5 & 1 & 0.5 & \cdots & 0.5 \\ \vdots & & \ddots & &\vdots \\ 0.5 & \cdots & 0.5 & 1 & 0.5 \\ 0.5 & \cdots & \cdots & 0.5 & 1 \\ \end{array} \right). $$
3. A block correlation structure between the covariates was considered, with a high correlation of *r*=0.7 between the biomarker *B* and a set of variables as well as between those variables, a moderate correlation of *r*=0.4 for another set and a correlation of *r*=0.1 or *r*=0 for the other variables:
  $$\Sigma_{3} = \left(\begin{array}{ccccccccccccc} 1 & 0.7 & 0.7 & 0.7 & 0.4 & 0.4 & 0.4 & 0.4 & 0.1 & 0.1 & 0.1 & 0 & 0 \\ 0.7 & 1 & 0.7 & 0.7 & 0.4 & 0.4 & 0.4 & 0.4 & 0.1 & 0.1 & 0.1 & 0 & 0 \\ 0.7 & 0.7 & 1 & 0.7 & 0.4 & 0.4 & 0.4 & 0.4 & 0.1 & 0.1 & 0.1 & 0 & 0 \\ 0.7 & 0.7 & 0.7 & 1 & 0.4 & 0.4 & 0.4 & 0.4 & 0.1 & 0.1 & 0.1 & 0 & 0 \\ 0.4 & 0.4 & 0.4 & 0.4 & 1 & 0.4 & 0.4 & 0.4 & 0.1 & 0.1 & 0.1 & 0 & 0 \\ 0.4 & 0.4 & 0.4 & 0.4 & 0.4 & 1 & 0.4 & 0.4 & 0.1 & 0.1 & 0.1 & 0 & 0 \\ 0.4 & 0.4 & 0.4 & 0.4 & 0.4 & 0.4 & 1 & 0.4 & 0.1 & 0.1 & 0.1 & 0 & 0 \\ 0.4 & 0.4 & 0.4 & 0.4 & 0.4 & 0.4 & 0.4 & 1 & 0.1 & 0.1 & 0.1 & 0 & 0 \\ 0.1 & 0.1 & 0.1 & 0.1 & 0.1 & 0.1 & 0.1 & 0.1 & 1 & 0.1 & 0.1 & 0 & 0 \\ 0.1 & 0.1 & 0.1 & 0.1 & 0.1 & 0.1 & 0.1 & 0.1 & 0.1 & 1 & 0.1 & 0 & 0 \\ 0.1 & 0.1 & 0.1 & 0.1 & 0.1 & 0.1 & 0.1 & 0.1 & 0.1 & 0.1 & 1 & 0 & 0 \\ 0.0 & 0.0 & 0.0 & 0.0 & 0.0 & 0.0 & 0.0 & 0.0 & 0.0 & 0.0 & 0.0 & 1 & 0 \\ 0.0 & 0.0 & 0.0 & 0.0 & 0.0 & 0.0 & 0.0 & 0.0 & 0.0 & 0.0 & 0.0 & 0 & 1 \end{array}\right). $$

For the scenarios with *K*=24 or *K*=36 potential predictors, the correlation matrices were adapted accordingly.

**Strength of association between prognostic variables and outcome**

For the strength of association between the potential prognostic variables *X*_1_,…,*X*_*K*_ and the outcome, two different settings were chosen:

1. For all covariates *X*_1_,…,*X*_*K*_, the same regression coefficient was chosen:
  $$\boldsymbol{\beta_{k}} = \boldsymbol{\beta_{eq}} = (\ln(1.1), \ldots, \ln(1.1))^{T} = (0.095, \ldots, 0.095)^{T}. $$
2. Varying strengths of association between the potential predictors and the risk for an event were considered. The vector of regression coefficients was chosen to be
  $$\boldsymbol{\beta_{k}} = \boldsymbol{\beta_{v}} = \left(\begin{array}{cc} \ln(1.2) \\ \ln(1.1)\\ \ln(1)\\ \ln(1.2)\\ \ln(1.1)\\ \ln(1)\\ \vdots\\ \ln(1.2)\\ \ln(1.1)\\ \ln(1) \end{array} \right) = \left(\begin{array}{cc} 0.182 \\ 0.095\\ 0\\ 0.182\\ 0.095\\ 0\\ \vdots\\ 0.182\\ 0.095\\ 0 \end{array} \right). $$

As all combinations of the different settings described above were considered in the simulation study, a total of 2 censoring distributions × 3 strengths of interaction between biomarker *B* and treatment *T* × 3 numbers of potential prognostic variables × 3 different correlation structures × 2 settings for association between the potential prognostics variables and the outcome = 108 settings were considered in the simulation. For each of these settings, 1000 simulation runs were performed.

### Analysis and presentation of results

In each simulation run, all of the methods or strategies described in “Strategies for covariate inclusion” section were fitted or applied, respectively. Estimation of the regression coefficients from the Cox regression models was performed with the function *coxph* in the *survival* library [22] of the statistical software R [20]. For the variable selection based on the AIC criterion, the function *stepAIC* in the library *MASS* [23] was applied.

For each model in each simulation run, the estimated regression coefficient for the biomarker–treatment interaction term $\hat {\beta }_{T\times B}$ and its estimated variance as well as the *p* value of the Wald test for the null hypothesisv *H*_0_: *β*_*T*×*B*_=0 was saved. Additionally, a 95% confidence interval for *β*_*T*×*B*_ was estimated as

3 $$ \begin{aligned} 95\% \operatorname{ci} &= \left\lbrack \hat{\beta}_{T\times B} - \phi_{0.975}\,\sqrt{\widehat{\operatorname{var}}(\hat{\beta}_{T\times B})};\right.\\ &\quad\ \ \left.\hat{\beta}_{T\times B} + \phi_{0.975}\,\sqrt{\widehat{\operatorname{var}}(\hat{\beta}_{T\times B})} \right\rbrack, \end{aligned}  $$

where *ϕ*_0.975_ denotes the 97.5% quantile of the standard normal distribution and $\widehat {\operatorname {var}}(\hat {\beta }_{T\times B})$ is the estimated variance of the interaction coefficient obtained in the corresponding simulation run for the respective modelling approach. If the algorithm for numerical maximization of the partial log-likelihood did not converge, this information was saved. All results presented in ‘Results’ rely on only estimations for which the numerical optimization algorithm converged. The number of runs for which no result was returned is presented.

For each model and strategy, the confidence interval coverage, i.e. the fraction of simulation runs in which the estimated confidence interval for the biomarker–treatment interaction covered the true value, was derived. The proportion of simulation runs in which the null hypothesis was rejected and a statistically significant biomarker–treatment interaction was detected for the conventional significance level of 5%, i.e. the power of the statistical test if H_0_ were false or the probability of a type I error if H_0_ were true (*β*_*T*×*B*_=0), was determined [24].

## Results

The observed proportions of rejected null hypotheses are summarized in Table 1. Results are presented stratified for different values of *K*, strength of interaction and proportion of censored observations, but were aggregated over different values of ***β***_***k***_ and ***Σ***. In Tables 2, 3 and 4, the observed proportions of simulation runs with rejected null hypotheses are shown separately for the scenarios with *K*=12 (Table 2), *K*=24 (Table 3) and *K*=36 (Table 4), for scenarios with no true biomarker–treatment interaction (top), true quantitative biomarker–treatment interaction (middle) and true qualitative biomarker–treatment interaction (bottom). An observed type I error probability of 7% was considered to be acceptable. For scenarios with no interaction (***β***_***T×B***_=0), observed type I error proportions larger than 7% are in italics. For scenarios with data generated under *H*_1_ (quantitative interaction and qualitative interaction), the proportions of rejected null hypotheses are in bold if the type I error probability for the approach at the given scenario was not larger than 7%.

**Table 1:** Proportions of rejected null hypotheses and numbers of included covariates stratified for number of potential prognostic variables (*K*), strength of interaction and proportion of censored observations

*K*	Interaction	Censoring	Main	True	AI*C*_*A*_	AI*C*_*B*_	Significance	Full
12	No	Low	0.058	0.060	0.062	0.056	0.059	0.060
12	No	High	0.054	0.051	0.056	0.048	0.053	0.054
12	Quantitative	Low	**0.118**	**0.133**	**0.139**	**0.131**	**0.130**	**0.134**
12	Quantitative	High	**0.095**	**0.100**	**0.108**	**0.099**	**0.096**	**0.099**
12	Qualitative	Low	**0.579**	**0.663**	**0.663**	**0.654**	**0.653**	**0.661**
12	Qualitative	High	**0.393**	**0.437**	**0.441**	**0.424**	**0.432**	**0.436**
24	No	Low	0.065	0.058	0.067	0.056	0.057	0.058
24	No	High	0.057	0.062	*0.073*	0.054	0.059	0.063
24	Quantitative	Low	**0.115**	**0.135**	**0.150**	**0.131**	**0.131**	**0.136**
24	Quantitative	High	**0.094**	**0.100**	0.114	**0.092**	**0.100**	**0.103**
24	Qualitative	Low	**0.465**	**0.634**	**0.645**	**0.616**	**0.610**	**0.633**
24	Qualitative	High	**0.349**	**0.411**	0.426	**0.383**	**0.399**	**0.415**
36	No	Low	0.065	0.059	*0.078*	0.056	0.061	0.063
36	No	High	0.067	0.068	*0.085*	0.057	0.066	*0.071*
36	Quantitative	Low	**0.114**	**0.132**	0.153	**0.118**	**0.130**	**0.134**
36	Quantitative	High	**0.093**	**0.102**	0.127	**0.093**	**0.101**	0.108
36	Qualitative	Low	**0.412**	**0.618**	0.629	**0.578**	**0.576**	**0.610**
36	Qualitative	High	**0.302**	**0.406**	0.431	**0.367**	**0.382**	0.402

Results are aggregated over different values of ***β***_***k***_ and ***Σ***. For the scenarios with no true biomarker–treatment interaction, results for methods/strategies with an observed type I error probability above 7% are in italics. For scenarios with a true biomarker–treatment interaction, the observed power is in bold if the type I error probability did not exceed 7%

**Table 2:** Proportions of rejected null hypotheses and numbers of included covariates for scenarios with *K*=12

*K*	*Σ*	*β*_*k*_	Censoring	Main	True	AI*C*_*A*_	AI*C*_*B*_	Significance	Full
No interaction									
12	*Σ*_1_	*β*_*eq*_	Low	0.066	0.069	*0.072*	0.066	0.067	0.069
12	*Σ*_1_	*β*_*v*_	Low	0.051	0.059	0.059	0.055	0.052	0.058
12	*Σ*_2_	*β*_*eq*_	Low	0.050	0.051	0.054	0.048	0.051	0.051
12	*Σ*_2_	*β*_*v*_	Low	0.060	0.047	0.052	0.047	0.052	0.052
12	*Σ*_3_	*β*_*eq*_	Low	0.060	0.065	0.066	0.061	0.063	0.065
12	*Σ*_3_	*β*_*v*_	Low	0.063	0.066	0.067	0.060	0.067	0.065
12	*Σ*_1_	*β*_*eq*_	High	0.057	0.056	0.060	0.054	0.055	0.056
12	*Σ*_1_	*β*_*v*_	High	0.038	0.040	0.037	0.034	0.040	0.044
12	*Σ*_2_	*β*_*eq*_	High	0.057	0.046	0.054	0.044	0.046	0.046
12	*Σ*_2_	*β*_*v*_	High	0.060	0.047	0.058	0.049	0.055	0.055
12	*Σ*_3_	*β*_*eq*_	High	0.053	0.059	0.060	0.050	0.062	0.059
12	*Σ*_3_	*β*_*v*_	High	0.057	0.056	0.064	0.055	0.061	0.065
Quantitative interaction									
12	*Σ*_1_	*β*_*eq*_	Low	**0.122**	**0.137**	0.143	**0.121**	**0.123**	**0.137**
12	*Σ*_1_	*β*_*v*_	Low	**0.119**	**0.134**	**0.139**	**0.132**	**0.134**	**0.136**
12	*Σ*_2_	*β*_*eq*_	Low	**0.105**	**0.131**	**0.136**	**0.129**	**0.131**	**0.131**
12	*Σ*_2_	*β*_*v*_	Low	**0.113**	**0.131**	**0.141**	**0.135**	**0.132**	**0.132**
12	*Σ*_3_	*β*_*eq*_	Low	**0.129**	**0.146**	**0.148**	**0.146**	**0.141**	**0.146**
12	*Σ*_3_	*β*_*v*_	Low	**0.121**	**0.121**	**0.127**	**0.120**	**0.116**	**0.120**
12	*Σ*_1_	*β*_*eq*_	High	**0.098**	**0.109**	**0.120**	**0.109**	**0.100**	**0.109**
12	*Σ*_1_	*β*_*v*_	High	**0.108**	**0.112**	**0.118**	**0.111**	**0.108**	**0.111**
12	*Σ*_2_	*β*_*eq*_	High	**0.077**	**0.088**	**0.099**	**0.086**	**0.088**	**0.088**
12	*Σ*_2_	*β*_*v*_	High	**0.104**	**0.095**	**0.106**	**0.096**	**0.093**	**0.093**
12	*Σ*_3_	*β*_*eq*_	High	**0.086**	**0.093**	**0.095**	**0.091**	**0.088**	**0.093**
12	*Σ*_3_	*β*_*v*_	High	**0.095**	**0.101**	**0.107**	**0.098**	**0.100**	**0.101**
Qualitative interaction									
12	*Σ*_1_	*β*_*eq*_	Low	**0.625**	**0.685**	0.673	**0.662**	**0.644**	**0.685**
12	*Σ*_1_	*β*_*v*_	Low	**0.605**	**0.664**	**0.664**	**0.661**	**0.649**	**0.661**
12	*Σ*_2_	*β*_*eq*_	Low	**0.517**	**0.641**	**0.641**	**0.634**	**0.641**	**0.641**
12	*Σ*_2_	*β*_*v*_	Low	**0.521**	**0.646**	**0.648**	**0.643**	**0.644**	**0.644**
12	*Σ*_3_	*β*_*eq*_	Low	**0.621**	**0.678**	**0.686**	**0.673**	**0.680**	**0.678**
12	*Σ*_3_	*β*_*v*_	Low	**0.583**	**0.661**	**0.664**	**0.652**	**0.661**	**0.658**
12	*Σ*_1_	*β*_*eq*_	High	**0.427**	**0.462**	**0.464**	**0.446**	**0.433**	**0.462**
12	*Σ*_1_	*β*_*v*_	High	**0.424**	**0.438**	**0.447**	**0.432**	**0.440**	**0.443**
12	*Σ*_2_	*β*_*eq*_	High	**0.338**	**0.403**	**0.410**	**0.389**	**0.403**	**0.403**
12	*Σ*_2_	*β*_*v*_	High	**0.359**	**0.431**	**0.429**	**0.413**	**0.433**	**0.433**
12	*Σ*_3_	*β*_*eq*_	High	**0.394**	**0.424**	**0.425**	**0.407**	**0.420**	**0.424**
12	*Σ*_3_	*β*_*v*_	High	**0.418**	**0.466**	**0.471**	**0.456**	**0.465**	**0.449**
Mean number of prognostic covariates included									
		*β*_*eq*_	Low	0	12	6.8	7.3	8.7	12
		*β*_*v*_	Low	0	8	6.4	6.9	8.7	12
		*β*_*eq*_	High	0	12	5.1	5.7	8.0	12
		*β*_*v*_	High	0	8	5.3	5.8	8.2	12

For the scenarios with no true biomarker–treatment interaction, results for methods/strategies with an observed type I error probability above 7% are in italics. For scenarios with a true biomarker–treatment interaction, the observed power is in bold if the type I error probability did not exceed 7%

**Table 3:** Proportions of rejected null hypotheses and numbers of included covariates for scenarios with *K*=24

*K*	*Σ*	*β*_*k*_	Censoring	Main	True	AI*C*_*A*_	AI*C*_*B*_	Significance	Full
No interaction									
24	*Σ*_1_	*β*_*eq*_	Low	0.044	0.049	0.065	0.053	0.048	0.049
24	*Σ*_1_	*β*_*v*_	Low	0.055	0.069	*0.074*	0.065	0.060	0.069
24	*Σ*_2_	*β*_*eq*_	Low	*0.087*	0.052	0.063	0.046	0.052	0.052
24	*Σ*_2_	*β*_*v*_	Low	0.068	*0.071*	*0.081*	0.066	*0.071*	*0.071*
24	*Σ*_3_	*β*_*eq*_	Low	0.068	0.049	0.061	0.052	0.055	0.049
24	*Σ*_3_	*β*_*v*_	Low	0.066	0.056	0.061	0.053	0.054	0.056
24	*Σ*_1_	*β*_*eq*_	High	0.035	0.056	0.069	0.054	0.046	0.056
24	*Σ*_1_	*β*_*v*_	High	0.051	*0.071*	*0.076*	0.059	0.068	*0.076*
24	*Σ*_2_	*β*_*eq*_	High	*0.073*	0.066	*0.074*	0.056	0.066	0.066
24	*Σ*_2_	*β*_*v*_	High	0.060	0.054	0.066	0.048	0.056	0.056
24	*Σ*_3_	*β*_*eq*_	High	0.062	0.058	*0.073*	0.047	0.059	0.058
24	*Σ*_3_	*β*_*v*_	High	0.059	0.068	*0.079*	0.060	0.057	0.066
Quantitative interaction									
24	*Σ*_1_	*β*_*eq*_	Low	**0.114**	**0.142**	**0.158**	**0.137**	**0.122**	**0.142**
24	*Σ*_1_	*β*_*v*_	Low	**0.106**	**0.138**	0.150	**0.132**	**0.125**	**0.143**
24	*Σ*_2_	*β*_*eq*_	Low	0.119	**0.135**	**0.148**	**0.130**	**0.135**	**0.135**
24	*Σ*_2_	*β*_*v*_	Low	**0.111**	0.124	0.145	**0.117**	0.126	0.126
24	*Σ*_3_	*β*_*eq*_	Low	**0.121**	**0.136**	**0.145**	**0.128**	**0.141**	**0.136**
24	*Σ*_3_	*β*_*v*_	Low	**0.121**	**0.136**	**0.157**	**0.141**	**0.134**	**0.136**
24	*Σ*_1_	*β*_*eq*_	High	**0.088**	**0.100**	**0.117**	**0.091**	**0.093**	**0.100**
24	*Σ*_1_	*β*_*v*_	High	**0.085**	0.104	0.110	**0.096**	**0.094**	0.111
24	*Σ*_2_	*β*_*eq*_	High	0.094	**0.109**	0.122	**0.094**	**0.109**	**0.109**
24	*Σ*_2_	*β*_*v*_	High	**0.113**	**0.096**	**0.116**	**0.088**	**0.100**	**0.100**
24	*Σ*_3_	*β*_*eq*_	High	**0.082**	**0.098**	0.109	**0.097**	**0.103**	**0.098**
24	*Σ*_3_	*β*_*v*_	High	**0.100**	**0.091**	0.109	**0.086**	**0.098**	**0.098**
Qualitative interaction									
24	*Σ*_1_	*β*_*eq*_	Low	**0.630**	**0.697**	**0.686**	**0.658**	**0.632**	**0.697**
24	*Σ*_1_	*β*_*v*_	Low	**0.547**	**0.678**	0.688	**0.656**	**0.615**	**0.685**
24	*Σ*_2_	*β*_*eq*_	Low	0.349	**0.610**	**0.619**	**0.595**	**0.610**	**0.610**
24	*Σ*_2_	*β*_*v*_	Low	**0.358**	0.590	0.608	**0.584**	0.578	0.578
24	*Σ*_3_	*β*_*eq*_	Low	**0.443**	**0.596**	**0.620**	**0.582**	**0.587**	**0.596**
24	*Σ*_3_	*β*_*v*_	Low	**0.465**	**0.632**	**0.651**	**0.621**	**0.636**	**0.631**
24	*Σ*_1_	*β*_*eq*_	High	**0.448**	**0.457**	**0.463**	**0.424**	**0.412**	**0.457**
24	*Σ*_1_	*β*_*v*_	High	**0.384**	0.453	0.466	**0.425**	**0.420**	0.464
24	*Σ*_2_	*β*_*eq*_	High	0.276	**0.364**	0.387	**0.340**	**0.364**	**0.364**
24	*Σ*_2_	*β*_*v*_	High	**0.292**	**0.397**	**0.423**	**0.378**	**0.411**	**0.411**
24	*Σ*_3_	*β*_*eq*_	High	**0.355**	**0.387**	0.405	**0.356**	**0.382**	**0.387**
24	*Σ*_3_	*β*_*v*_	High	**0.338**	**0.408**	0.409	**0.377**	**0.404**	**0.408**
Mean number of prognostic covariates included									
		*β*_*eq*_	Low	0	24	13.2	13.7	17.5	24
		*β*_*v*_	Low	0	16	12.7	13.1	17.8	24
		*β*_*eq*_	High	0	24	10.2	10.7	16.5	24
		*β*_*v*_	High	0	16	10.6	11.0	16.8	24

For the scenarios with no true biomarker–treatment interaction, results for methods/strategies with an observed type I error probability above 7% are in italics. For scenarios with a true biomarker–treatment interaction, the observed power is in bold if the type I error probability did not exceed 7%

**Table 4:** Proportions of rejected null hypotheses and numbers of included covariates for scenarios with *K*=36

*K*	*Σ*	*β*_*k*_	Censoring	Main	True	AI*C*_*A*_	AI*C*_*B*_	Significance	Full
No interaction									
36	*Σ*_1_	*β*_*eq*_	Low	0.047	0.065	*0.080*	0.054	0.056	0.065
36	*Σ*_1_	*β*_*v*_	Low	0.059	0.067	*0.084*	0.066	0.069	*0.073*
36	*Σ*_2_	*β*_*eq*_	Low	*0.077*	0.053	*0.073*	0.051	0.053	0.053
36	*Σ*_2_	*β*_*v*_	Low	*0.075*	0.054	*0.074*	0.052	0.056	0.056
36	*Σ*_3_	*β*_*eq*_	Low	0.067	0.060	*0.083*	0.057	0.064	0.060
36	*Σ*_3_	*β*_*v*_	Low	0.063	0.055	*0.074*	0.053	0.069	0.069
36	*Σ*_1_	*β*_*eq*_	High	0.052	*0.071*	*0.086*	0.059	0.055	*0.071*
36	*Σ*_1_	*β*_*v*_	High	0.047	0.063	*0.080*	0.058	0.050	0.066
36	*Σ*_2_	*β*_*eq*_	High	*0.085*	*0.080*	*0.082*	0.057	*0.080*	*0.080*
36	*Σ*_2_	*β*_*v*_	High	*0.085*	0.064	*0.086*	0.054	0.070	0.070
36	*Σ*_3_	*β*_*eq*_	High	0.057	0.069	*0.094*	0.056	0.063	0.069
36	*Σ*_3_	*β*_*v*_	High	*0.075*	0.063	*0.081*	0.057	*0.076*	*0.071*
Quantitative interaction									
36	*Σ*_1_	*β*_*eq*_	Low	**0.103**	**0.150**	0.165	**0.130**	**0.138**	**0.150**
36	*Σ*_1_	*β*_*v*_	Low	**0.095**	**0.131**	0.150	**0.120**	**0.125**	0.136
36	*Σ*_2_	*β*_*eq*_	Low	0.121	**0.120**	0.141	**0.109**	**0.120**	**0.120**
36	*Σ*_2_	*β*_*v*_	Low	0.128	**0.128**	0.147	**0.121**	**0.134**	**0.134**
36	*Σ*_3_	*β*_*eq*_	Low	**0.115**	**0.121**	0.142	**0.103**	**0.122**	**0.121**
36	*Σ*_3_	*β*_*v*_	Low	**0.119**	**0.143**	0.172	**0.125**	**0.140**	**0.143**
36	*Σ*_1_	*β*_*eq*_	High	**0.081**	0.108	0.133	**0.098**	**0.094**	0.108
36	*Σ*_1_	*β*_*v*_	High	**0.095**	**0.112**	0.132	**0.101**	**0.102**	**0.118**
36	*Σ*_2_	*β*_*eq*_	High	0.100	0.085	0.103	**0.072**	0.085	0.085
36	*Σ*_2_	*β*_*v*_	High	0.092	**0.109**	0.127	**0.093**	**0.118**	**0.118**
36	*Σ*_3_	*β*_*eq*_	High	**0.091**	**0.104**	0.134	**0.099**	**0.105**	**0.104**
36	*Σ*_3_	*β*_*v*_	High	0.097	**0.093**	0.132	**0.093**	0.102	0.115
Qualitative interaction									
36	*Σ*_1_	*β*_*eq*_	Low	**0.551**	**0.652**	0.657	**0.603**	**0.558**	**0.652**
36	*Σ*_1_	*β*_*v*_	Low	**0.517**	**0.700**	0.688	**0.658**	**0.599**	0.669
36	*Σ*_2_	*β*_*eq*_	Low	0.280	**0.570**	0.582	**0.518**	**0.570**	**0.570**
36	*Σ*_2_	*β*_*v*_	Low	0.266	**0.555**	0.575	**0.517**	**0.542**	**0.542**
36	*Σ*_3_	*β*_*eq*_	Low	**0.408**	**0.609**	0.637	**0.582**	**0.581**	**0.609**
36	*Σ*_3_	*β*_*v*_	Low	**0.451**	**0.623**	0.637	**0.592**	**0.605**	**0.620**
36	*Σ*_1_	*β*_*eq*_	High	**0.389**	0.447	0.456	**0.403**	**0.385**	0.447
36	*Σ*_1_	*β*_*v*_	High	**0.390**	**0.472**	0.486	**0.437**	**0.418**	**0.453**
36	*Σ*_2_	*β*_*eq*_	High	0.219	0.368	0.411	**0.334**	0.368	0.368
36	*Σ*_2_	*β*_*v*_	High	0.228	**0.385**	0.419	**0.353**	**0.389**	**0.389**
36	*Σ*_3_	*β*_*eq*_	High	**0.282**	**0.364**	0.396	**0.328**	**0.352**	**0.364**
36	*Σ*_3_	*β*_*v*_	High	0.303	**0.402**	0.416	**0.350**	0.382	0.391
Mean number of prognostic covariates included									
		*β*_*eq*_	Low	0	36	19.6	19.9	24.5	36
		*β*_*v*_	Low	0	24	19.0	19.4	25.2	36
		*β*_*eq*_	High	0	36	15.2	15.7	23.1	36
		*β*_*v*_	High	0	24	16.0	16.4	23.9	36

For the scenarios with no true biomarker–treatment interaction, results for methods/strategies with an observed type I error probability above 7% are in italics. For scenarios with a true biomarker–treatment interaction, the observed power is in bold if the type I error probability did not exceed 7%

The mean numbers of included additional covariates are given for each method or strategy for sets of scenarios stratified for ***β***_***k***_ and amount of censoring in the bottom rows of Tables 2, 3 and 4 and for each of the 108 simulated scenarios in Additional file 7: Table S1 (for *K*=12), Additional file 8: Table S2 (for *K*=24) and Additional file 9: Table S3 (for *K*=36).

**Fig. 2:**
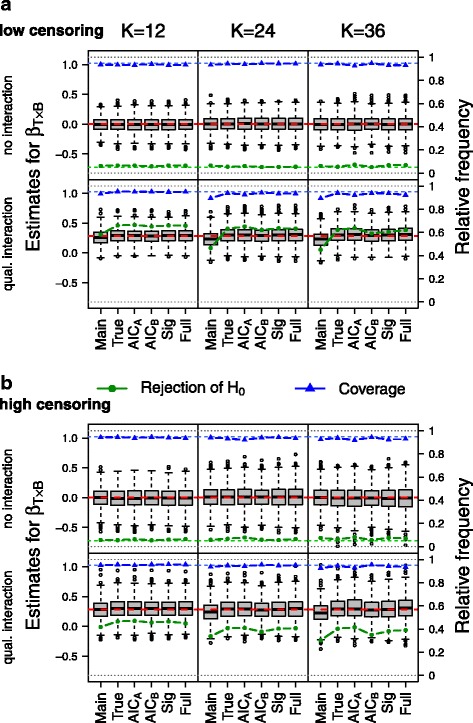
Distribution of $\hat {\beta }_{T\times B}$ for scenarios with *Σ*=*Σ*_3_, ***β***_***k***_ = ***β***_***v***_, and low censoring (**a**) or high censoring (**b**) for no biomarker–treatment interaction (*β*_*T*×*B*_= ln(1.0)=0, top rows) or qualitative biomarker–treatment interaction (*β*_*T*×*B*_= ln(1.33)=0.285, bottom rows). Scenarios for different numbers of potential prognostic variables are shown in different columns. The dashed red lines indicate the true value of *β*_*T*×*B*_, the blue triangles represent the observed confidence interval coverages and the green dots the observed probability for a type I error (**a**) or estimated power (**b**). AIC Akaike’s information criterion, qual. qualitative, Sig significance

The distributions of the obtained estimates are illustrated in Fig. 2 for one exemplary set of scenarios. The observed distributions of the regression coefficient estimates for the biomarker–treatment interaction $\hat {\beta }_{T\times B}$ are displayed as box plots for the scenarios with ***Σ***=***Σ***_***3***_, ***β***_***k***_***=β***_***v***_ and low (a) or high number of censored observations (b). In the top rows, scenarios with no true biomarker–treatment interaction are shown, and in the bottom rows, results for data simulated with true qualitative biomarker–treatment interactions are presented. Scenarios with different numbers of (potential) prognostic variables (*K*=12, *K*=24 and *K*=36) are shown in separate columns. Distributions of estimated regression coefficients are illustrated for all scenarios with no true interaction (under *H*_0_) or with true qualitative interaction in Additional file 1: Figure S1, Additional file 2: Figure S2, Additional file 3: Figure S3, Additional file 4: Figure S4, Additional file 5: Figure S5 and Additional file 6: Figure S6. In each figure, the true value of the interaction regression coefficient is illustrated by the horizontal red line. Additionally, the confidence interval coverage for each modelling strategy (triangles and blue lines) and the probability of rejection of the null hypothesis of no biomarker–treatment interaction, i.e. the estimated probability for a type I error in the first row and the observed power in the second row, are illustrated (circles and green lines).

The type I error probabilities for the biomarker–treatment interaction term, which are presented in the lines indicated with no interaction (Table 1) and in the upper parts of Tables 2, 3 and 4 for the scenarios with no interaction, were acceptable for almost all methods and strategies, when *K*=12 further (potential) prognostic variables were considered. Only for strategy AIC _*A*_ an unacceptably high probability of type I errors (defined as larger than 7%) was observed for one setting (Table 2). For scenarios with *K*=24 (potential) prognostic variables, increased type I error probabilities were observed for each method for at least one scenario, except for AIC _*B*_. For AIC _*A*_, type I error probabilities above 7% were observed for six of the 12 settings (Table 3) and for scenarios with a high proportion of censored observations (60% to 70%) when scenarios with different ***β***_***k***_ and ***Σ*** were aggregated (Table 1). When *K* = 36 potential predictors were considered, an increased type I error probability was observed for AIC _*A*_ for all scenarios. For main, significance and full, elevated false positive rates were obtained for three to five scenarios with a high proportion of censored observations. For the true model, only two scenarios with a high proportion of censored observations led to rejection of the null hypothesis in more than 7% of the observed simulation runs (***Σ***_***2***_, ***β***_***eq***_ and ***Σ***_***1***_, ***β***_***eq***_). For all other scenarios, the observed type I error probabilities were between 5% and 7%. For the strategy AIC _*B*_, all observed type I error probabilities were between 5% and 7%.

For the main model, regression coefficients for the biomarker–treatment effect were underestimated when a true biomarker–treatment interaction was present (Fig. 2), with the largest bias observed for scenarios with ***Σ***=***Σ***_***2***_ (see second rows of Additional file 1: Figure S1A and Figure S1B, Additional file 2: Figure S2A and Figure S2B, Additional file 3: Figure S3A and Figure S3B, Additional file 4: Figure S4A and Figure S4B, Additional file 5: Figure S5A and Figure S5B, and Additional file 6: Figure S6A and Figure S6B). This also led to a loss of power, which was reduced as compared to the true model for most of the scenarios (Tables 1, 2, 3 and 4, green dots in Additional file 1: Figure S1, Additional file 2: Figure S2, Additional file 3: Figure S3, Additional file 4: Figure S4, Additional file 5: Figure S5 and Additional file 6: Figure S6). Generally, the highest power was observed for the true model. The power for AIC _*A*_ cannot be interpreted adequately for most of the scenarios due to its increased type I error probabilities. The full model is identical to the true model for ***β***_***k***_=***β***_***eq***_, as all covariates are truly associated with the outcome. For ***β***_***k***_=***β***_***v***_, the power of the full model was similar to the power of the true model for *K*=12 and *K*=24 in our simulation runs, but was slightly lower for simulations with *K*=36. The strategy AIC _*B*_, which appears to have an adequate false positive rate, showed (slightly) lower power than the true model for (almost) all of the scenarios. A slightly decreased power was also observed for the strategy including all covariates that were significantly associated with the outcome (significance). The type I error probability was acceptable for most scenarios with a small or moderate number of potential predictors (*K*=12 and *K*=24), but an increased type I error probability was observed for scenarios with many potential predictors (*K*=36).

Coverage was adequate for most of the models and strategies. For main, the coverage was reduced for some scenarios due to biased estimates. For AIC _*A*_, the coverage was under 93% for 52 of the 108 scenarios (48.1*%*), indicating standard errors for the regression coefficient of interest were underestimated following the variable selection procedure.

In the last rows of Tables 2, 3 and 4, the mean numbers of additionally included covariates are summarized for each method/strategy stratified for the amount of censoring and ***β***_***k***_ (which determines the number of truly prognostic variables). It was observed that for our settings, the procedure including variables that were significantly associated with the outcome in univariate Cox models selected more variables than the AIC-based methods, and that slightly more variables were chosen with AIC _*B*_ than with AIC _*A*_. For scenarios with ***β***_***k***_=***β***_***eq***_, the true and full models were identical by definition. More detailed information on the numbers of covariates included are given in Additional file 7: Table S1, Additional file 8: Table S2 and Additional file 9: Table S3.

The optimization algorithm for numerical maximization of the partial log-likelihood of the Cox regression model for estimating the regression coefficients did not converge for some simulation runs. The problem especially occurred for AIC _*A*_. Over all 108,000 simulation runs (108 scenarios × 1,000 runs per scenario), the estimation algorithm did not converge 11 times (0.010*%*) for main, twice (0.002*%*) for true, 895 times (0.829*%*) for AIC _*A*_, 27 times (0.025*%*) for AIC _*B*_, three times (0.003*%*) for significance and no times (0%) for full.

## Discussion

The ultimate goal in individualized or tailored medicine is to find the best treatment for each individual based on the patient’s characteristics like age, sex, co-morbidities, disease history and molecular and genetic information, which are often referred to as biomarkers. The existence and detection of a biomarker–treatment interaction can be considered as a requirement for such treatment individualization [2], and consequently an interaction between the biomarker of interest and treatment has to be established in a first step, e.g. by finding statistically significant and clinically relevant interactions based on data from (multiple) randomized clinical trials. Decision rules for treatment selection based on the characteristics of a certain patient have to be investigated and established afterwards, also considering the benefits and costs of the application of a certain treatment strategy for a given patient.

To detect relevant associations and interactions, it is well known that splitting a quantitative variable into different categories, leading to a comparison of treatment effects between different subgroups, will result in a loss of information and will consequently decrease the probability of detecting a true biomarker–treatment interaction [25]. So, using all the quantitative information is recommended for analysis of biomarker–treatment interactions [7]. To estimate a treatment effect in a randomized clinical trial, the inclusion of relevant prognostic variables is recommended [10] to increase the precision of the estimate and consequently the probability of detecting real group differences. For this article, we performed a simulation study to investigate whether the probability of detecting a biomarker–treatment interaction in data derived from a randomized clinical trial can be improved by including further potentially prognostic variables in a Cox regression model for time-to-event data. Different settings for the strength of interaction between the biomarker and the treatment, the correlation between the biomarker of interest and other potential predictors, the strength of association between the predictors and outcome, the number of (potential) further predictors, and the number of events and censored observations were considered. When a biomarker–treatment interaction is assessed using data from a randomized clinical trial, obviously the best choice is to include in the final model all covariates truly associated with the outcome, which was covered by the true model in our simulation study. As this true model often is not known in practice, especially in investigations including molecular or genetic information, more flexible approaches might be needed. So, we also investigated strategies using data-driven variable selection procedures based on AIC [14] or on the results of Cox regression models with single covariates.

In our simulation study, we observed that including the correct prognostic variables leads to an increased probability of detecting a true biomarker–treatment interaction and reduced bias of the estimated interaction effect, with the magnitude of improvement depending on the strength of association between the prognostic variables and the outcome and between the prognostic variables and the biomarker of interest. In contrast, including too many variables per event can lead to the opposite effect and increased probabilities of false positives. This problem is well known for multiple regression models [15, 26]. Our results support the rule of ten, which was proposed for predictive modelling [27], since the type I error probability was increased for the biomarker of interest, even for the true model, when a large number of covariates was considered. The simulation study also revealed that ignoring relevant prognostic factors leads to biased estimates for the biomarker–treatment interaction effect, which has been described for estimating the group effect from a randomized clinical trial using a Cox regression model [11]. Generally, the data-driven selection of prognostic variables by an inclusion procedure based on the AIC after including the main effects of the biomarker of interest, the treatment and their interaction in the model increases the type I error probabilities and reduces the confidence interval coverage. This was not observed in a strategy that selected the relevant prognostic variables in a first step and added the biomarker main effect and the biomarker–treatment interaction afterwards (called AIC _*B*_ in our article). In our simulated scenarios, the strategy including all covariates that were found to be significantly associated with the outcome performed similarly to that approach. Automated variable selection procedures are criticized in the literature for various reasons (see e.g. [28]). Based on the results of our simulation study, we strongly discourage using an automated variable selection procedure to choose additional prognostic variables after including the biomarker–treatment interaction of interest, as this may lead to unreliable results.

An obvious limitation of our study is that we observed only a moderate number of different scenarios with three correlation structures, three strengths of interaction between the biomarker and treatment, two strengths/structures of association between the additional prognostic variables and treatment, two censoring distributions, three numbers of (potential) prognostic variables, and a fixed number of 500 observations, due to limited time and space. All these aspects influenced the results and other settings may have led to different findings and consequently recommendations. In particular, the number of observed events, which is more important than the total sample size for a time-to-event outcome, was varied only by choosing two different censoring proportions, but it has a major impact on the power of the interaction test. We also investigated only a small number of strategies for inclusion or selection of further covariates based on the AIC and significant associations with the outcome. Other strategies (like backward selection), other criteria (like the Bayesian information criterion [29]) or other procedures for variable selection (like the least absolute shrinkage and selection operator [30]) were not considered. Furthermore, we considered only normally distributed biomarkers and linear associations and interactions in our simulations and fitted Cox regression models assuming linear associations and time-constant effects to our data. Recently introduced methods for estimating non-linear interactions, like local partial likelihood estimation [31], multivariable fractional polynomials for interaction [8] or the modified covariate approach [9], were not investigated.

It has to be considered that in our scenario, only one pre-specified biomarker of interest is assessed. It was identified as being of interest e.g. in an observational study or was found to be relevant for a similar kind of disease. If more than one biomarker is investigated, multiplicity issues arise that have to be adequately considered [32]. When an analysis is an additional analysis to a standard group comparison for a randomized clinical trial, it can only be exploratory in nature. Nevertheless, the method used for statistical analysis should be specified a priori to generate reliable results and avoid problems of data-dredging and selective reporting, and consequently generating unreliable results and increased false-positive rates [33]. Further algorithms or strategies should be used in sensitivity analyses to assess the stability of the observed results. If the investigation of a biomarker–treatment interaction is of major importance for a clinical trial, this should be considered in the design stage and consequently in the sample size calculation.

## Conclusions

Based on the results of our simulation study, we recommend considering prognostic covariates in regression models when estimating biomarker–treatment interactions, as the power for detecting true interactions can be increased. However, including too many variables can lead to unreliable results. The choice of variables included should be based on prior information and subject knowledge. Automatic variable selection procedures have to be handled with care.

## Additional files

Additional file 1**Figure S1**. Distribution of $\hat {\beta }_{T\times B}$ for scenarios with *K*=12, ***β***_***k***_ = ***β***_***eq***_, and low censoring (A) or high censoring (B) for no biomarker–treatment interaction (*β*_*T*×*B*_= ln(1.0)=0, top rows) or qualitative biomarker–treatment interaction (*β*_*T*×*B*_= ln(1.33)=0.285, bottom rows). Results for different correlation structures are shown in separate columns. The dashed red lines indicate the true value of *β*_*T*×*B*_, the blue triangles represent the observed confidence interval coverages, the green dots the observed probability for a type I error (A) or estimated power (B). (PDF 20 kb)

Additional file 2**Figure S2**. Distribution of $\hat {\beta }_{T\times B}$ for scenarios with *K*=12, ***β***_***k***_ = ***β***_***v***_, and low censoring (A) or high censoring (B) for no biomarker–treatment interaction (*β*_*T*×*B*_= ln(1.0)=0, top rows) or qualitative biomarker–treatment interaction (*β*_*T*×*B*_= ln(1.33)=0.285, bottom rows). Results for different correlation structures are shown in separate columns. The dashed red lines indicate the true value of *β*_*T*×*B*_, the blue triangles represent the observed confidence interval coverages, the green dots the observed probability for a type I error (A) or estimated power (B). (PDF 20 kb)

Additional file 3**Figure S3**. Distribution of $\hat {\beta }_{T\times B}$ for scenarios with *K*=24, ***β***_***k***_ = ***β***_***eq***_, and low censoring (A) or high censoring (B) for no biomarker–treatment interaction (*β*_*T*×*B*_= ln(1.0)=0, top rows) or qualitative biomarker–treatment interaction (*β*_*T*×*B*_= ln(1.33)=0.285, bottom rows). Results for different correlation structures are shown in separate columns. The dashed red lines indicate the true value of *β*_*T*×*B*_, the blue triangles represent the observed confidence interval coverages, the green dots the observed probability for a type I error (A) or estimated power (B). (PDF 20 kb)

Additional file 4**Figure S4**. Distribution of $\hat {\beta }_{T\times B}$ for scenarios with *K*=24, ***β***_***k***_ = ***β***_***v***_, and low censoring (A) or high censoring (B) for no biomarker–treatment interaction (*β*_*T*×*B*_= ln(1.0)=0, top rows) or qualitative biomarker–treatment interaction (*β*_*T*×*B*_= ln(1.33)=0.285, bottom rows). Results for different correlation structures are shown in separate columns. The dashed red lines indicate the true value of *β*_*T*×*B*_, the blue triangles represent the observed confidence interval coverages, the green dots the observed probability for a type I error (A) or estimated power (B). (PDF 20 kb)

Additional file 5**Figure S5**. Distribution of $\hat {\beta }_{T\times B}$ for scenarios with *K*=36, ***β***_***k***_ = ***β***_***eq***_, and low censoring (A) or high censoring (B) for no biomarker–treatment interaction (*β*_*T*×*B*_= ln(1.0)=0, top rows) or qualitative biomarker–treatment interaction (*β*_*T*×*B*_= ln(1.33)=0.285, bottom rows). Results for different correlation structures are shown in separate columns. The dashed red lines indicate the true value of *β*_*T*×*B*_, the blue triangles represent the observed confidence interval coverages, the green dots the observed probability for a type I error (A) or estimated power (B). (PDF 20 kb)

Additional file 6**Figure S6**. Distribution of $\hat {\beta }_{T\times B}$ for scenarios with *K*=36, ***β***_***k***_ = ***β***_***v***_, and low censoring (A) or high censoring (B) for no biomarker–treatment interaction (*β*_*T*×*B*_= ln(1.0)=0, top rows) or qualitative biomarker–treatment interaction (*β*_*T*×*B*_= ln(1.33)=0.285, bottom rows). Results for different correlation structures are shown in separate columns. The dashed red lines indicate the true value of *β*_*T*×*B*_, the blue triangles represent the observed confidence interval coverages, the green dots the observed probability for a type I error (A) or estimated power (B). (PDF 20 kb)

Additional file 7 **Table S1**. Mean number of additionally included prognostic variables for all scenarios with *K*=12. (PDF 68 kb)

Additional file 8 **Table S2**. Mean number of additionally included prognostic variables for all scenarios with *K*=24. (PDF 68 kb)

Additional file 9 **Table S3**. Mean number of additionally included prognostic variables for all scenarios with *K*=36. (PDF 68 kb)
