# Supplementary figures and images for: A simulation study on estimating biomarker–treatment interaction effects in randomized trials with prognostic variables
Source: Trials. 2018 Feb 20;19:128. doi: 10.1186/s13063-018-2491-0 (PMC5819679; doi:10.1186/s13063-018-2491-0)

low censoring

 $\Sigma_1$  $\Sigma_2$  $\Sigma_3$ 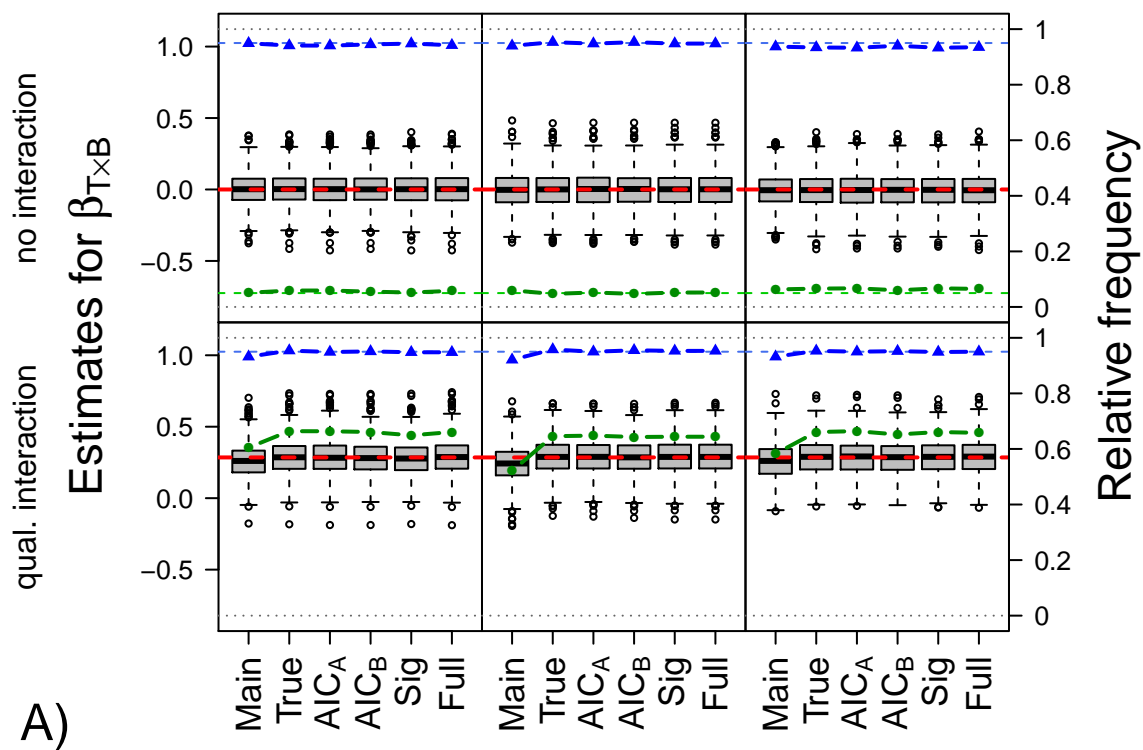

high censoring

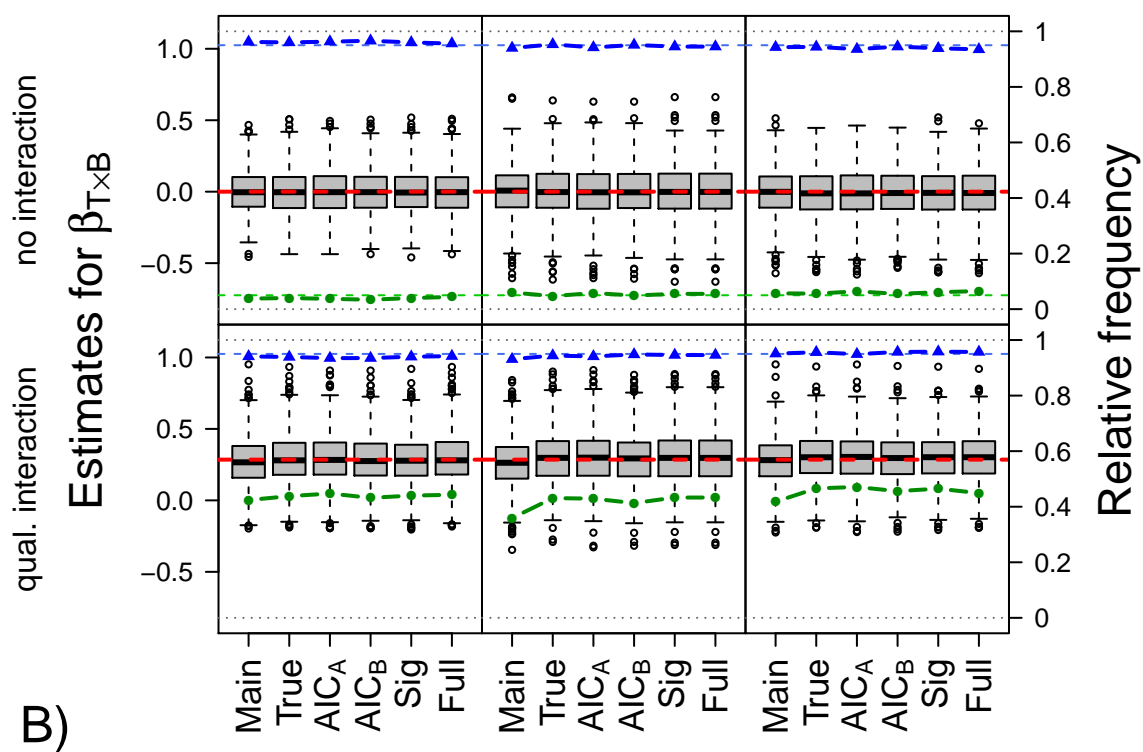

Supplement: Supplementary file 2 — Figure S2. Distribution of $\hat {\beta }_{T\times B}$β^T×B for scenarios with K=12, βk = βv, and low censoring (A) or high censoring (B) for no biomarker–treatment interaction (βT×B= ln(1.0)=0, top rows) or qualitative biomarker–treatment interaction (βT×B= ln(1.33)=0.285, bottom rows). Results for different correlation structures are shown in separate columns. The dashed red lines indicate the true value of βT×B, the blue triangles represent the observed confidence interval coverages, the green dots the observed probability for a type I error (A) or estimated power (B). (PDF 20 kb) [file 13063_2018_2491_MOESM2_ESM.pdf]

low censoring

$\Sigma_1$

$\Sigma_2$

$\Sigma_3$

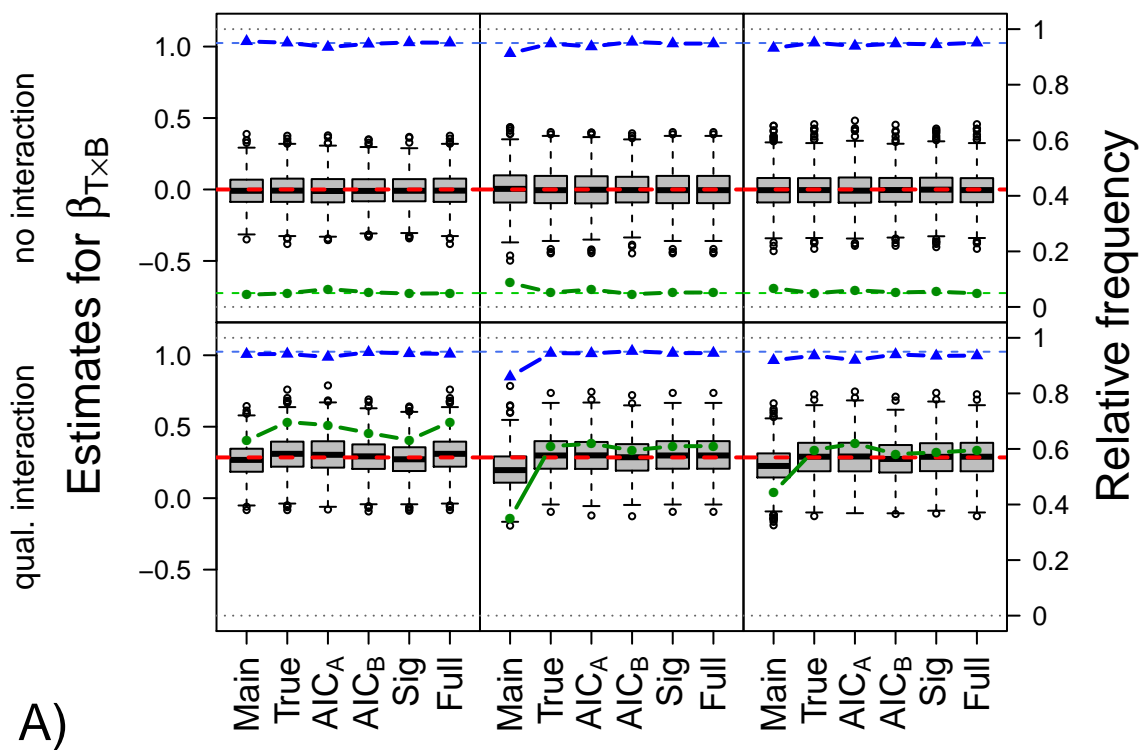

● Rejection of  $H_0$  ▲ Coverage

high censoring

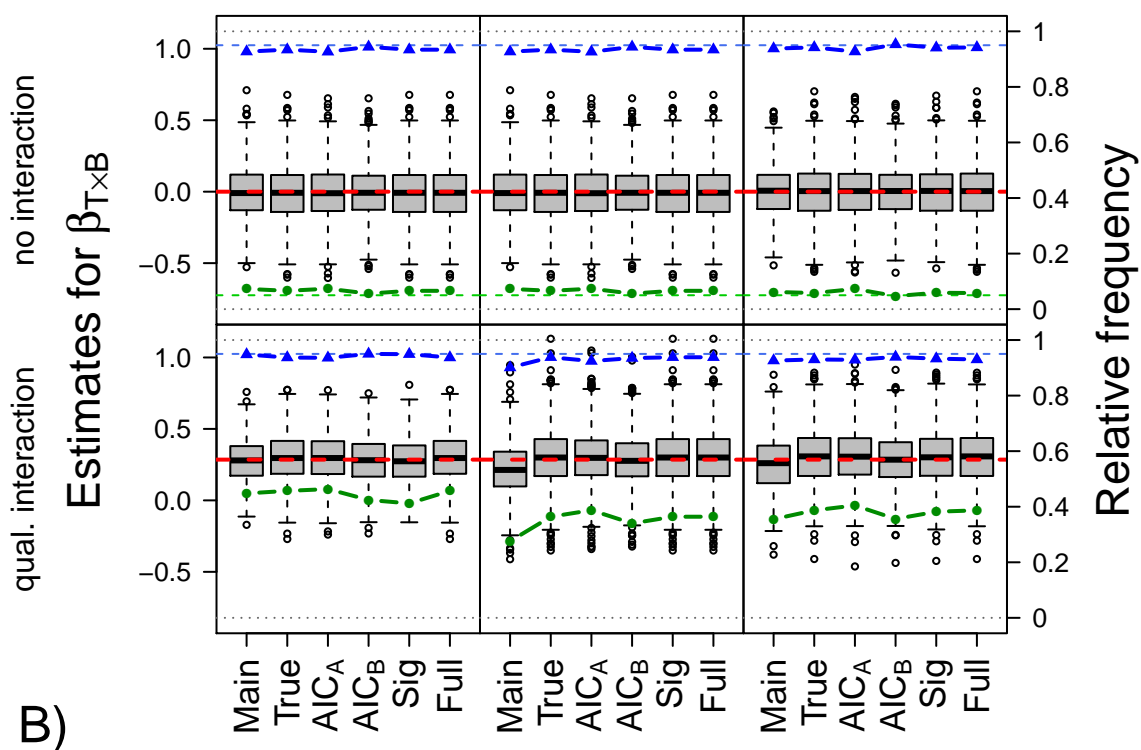

Supplement: Supplementary file 3 — Figure S3. Distribution of $\hat {\beta }_{T\times B}$β^T×B for scenarios with K=24, βk = βeq, and low censoring (A) or high censoring (B) for no biomarker–treatment interaction (βT×B= ln(1.0)=0, top rows) or qualitative biomarker–treatment interaction (βT×B= ln(1.33)=0.285, bottom rows). Results for different correlation structures are shown in separate columns. The dashed red lines indicate the true value of βT×B, the blue triangles represent the observed confidence interval coverages, the green dots the observed probability for a type I error (A) or estimated power (B). (PDF 20 kb) [file 13063_2018_2491_MOESM3_ESM.pdf]

low censoring

$\Sigma_1$

$\Sigma_2$

$\Sigma_3$

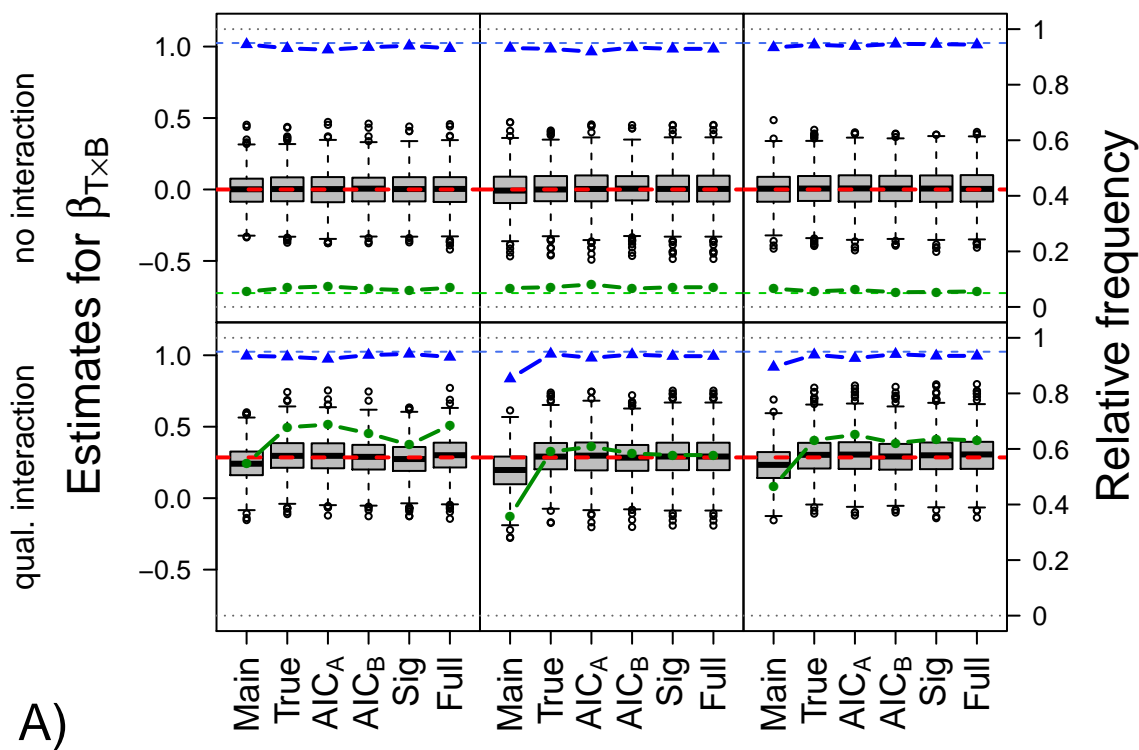

Rejection of  $H_0$

Coverage

high censoring

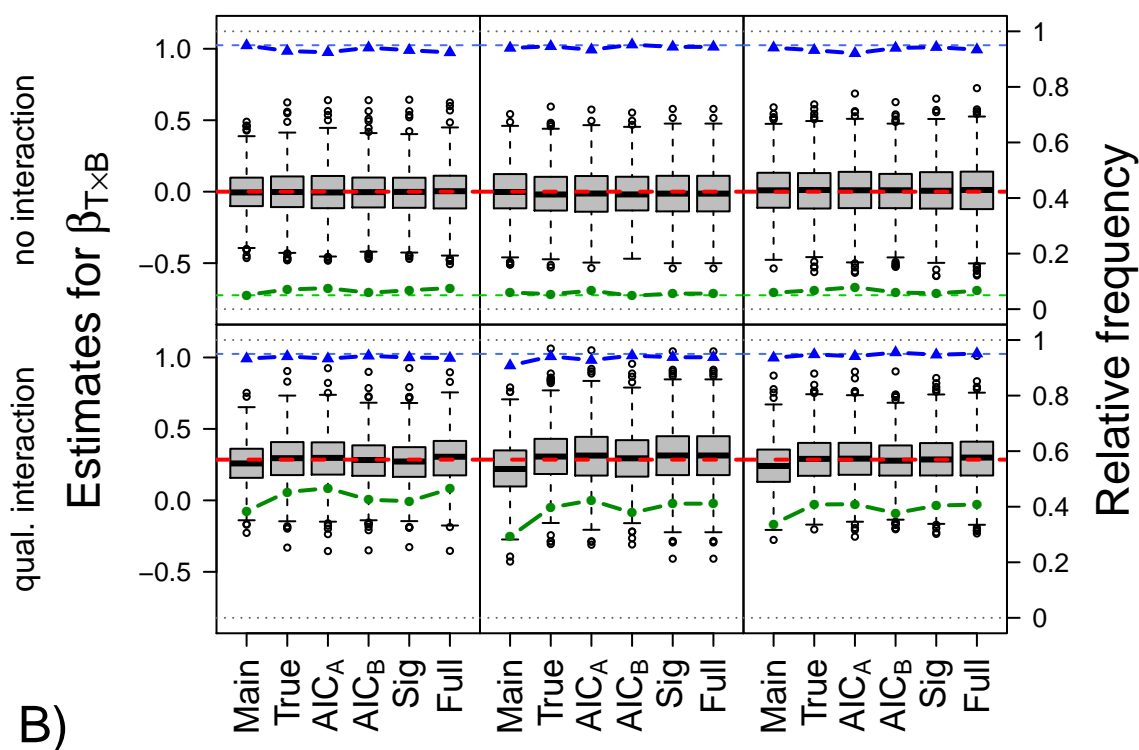

Supplement: Supplementary file 4 — Figure S4. Distribution of $\hat {\beta }_{T\times B}$β^T×B for scenarios with K=24, βk = βv, and low censoring (A) or high censoring (B) for no biomarker–treatment interaction (βT×B= ln(1.0)=0, top rows) or qualitative biomarker–treatment interaction (βT×B= ln(1.33)=0.285, bottom rows). Results for different correlation structures are shown in separate columns. The dashed red lines indicate the true value of βT×B, the blue triangles represent the observed confidence interval coverages, the green dots the observed probability for a type I error (A) or estimated power (B). (PDF 20 kb) [file 13063_2018_2491_MOESM4_ESM.pdf]
