# Supplementary material for: A simulation study on estimating biomarker–treatment interaction effects in randomized trials with prognostic variables
Source: Trials. 2018 Feb 20;19:128. doi: 10.1186/s13063-018-2491-0 (PMC5819679; doi:10.1186/s13063-018-2491-0)
Supplement: Supplementary file 5 — Figure S5. Distribution of $\hat {\beta }_{T\times B}$β^T×B for scenarios with K=36, βk = βeq, and low censoring (A) or high censoring (B) for no biomarker–treatment interaction (βT×B= ln(1.0)=0, top rows) or qualitative biomarker–treatment interaction (βT×B= ln(1.33)=0.285, bottom rows). Results for different correlation structures are shown in separate columns. The dashed red lines indicate the true value of βT×B, the blue triangles represent the observed confidence interval coverages, the green dots the observed probability for a type I error (A) or estimated power (B). (PDF 20 kb) [file 13063_2018_2491_MOESM5_ESM.pdf]

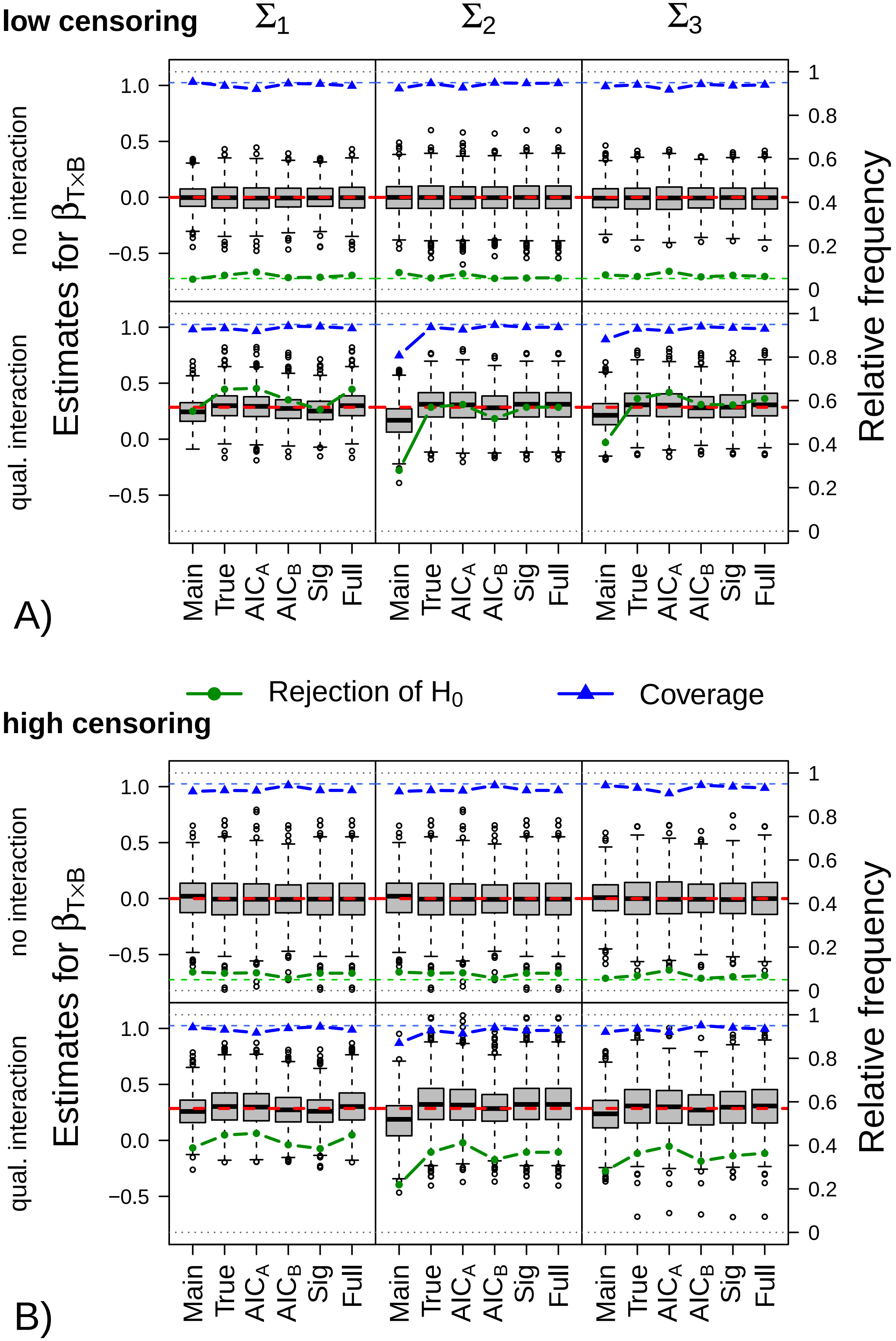

low censoring
Σ1
Σ2
Σ3
no interaction
qual. interaction
Estimates for βT×B
Relative frequency
Main
True
AICA
AICB
Sig
Full
A)
Rejection of H0
Coverage
high censoring
B)
