# Supplementary material for: A simulation study on estimating biomarker–treatment interaction effects in randomized trials with prognostic variables
Source: Trials. 2018 Feb 20;19:128. doi: 10.1186/s13063-018-2491-0 (PMC5819679; doi:10.1186/s13063-018-2491-0)
Supplement: Supplementary file 7 — Table S1. Mean number of additionally included prognostic variables for all scenarios with K=12. (PDF 68 kb) [file 13063_2018_2491_MOESM7_ESM.pdf]

K	$\boldsymbol{\Sigma}$	$\boldsymbol{\beta_k}$	**Interact.**	**Cens.**	**Main**	**True**	**$\mathbf{AIC_A}$**	**$\mathbf{AIC_B}$**	**Sig**	**Full**
12	$\boldsymbol{\Sigma_1}$	$\boldsymbol{\beta_{eq}}$	no	low	0	12	7.2	6.9	4.1	12
12	$\boldsymbol{\Sigma_2}$	$\boldsymbol{\beta_{eq}}$	no	low	0	12	6.4	7.3	12.0	12
12	$\boldsymbol{\Sigma_3}$	$\boldsymbol{\beta_{eq}}$	no	low	0	12	6.8	7.7	10.1	12
12	$\boldsymbol{\Sigma_1}$	$\boldsymbol{\beta_{eq}}$	quant.	low	0	12	7.1	6.8	3.9	12
12	$\boldsymbol{\Sigma_2}$	$\boldsymbol{\beta_{eq}}$	quant.	low	0	12	6.4	7.4	12.0	12
12	$\boldsymbol{\Sigma_3}$	$\boldsymbol{\beta_{eq}}$	quant.	low	0	12	6.8	7.8	10.0	12
12	$\boldsymbol{\Sigma_1}$	$\boldsymbol{\beta_{eq}}$	qual.	low	0	12	7.1	6.6	3.7	12
12	$\boldsymbol{\Sigma_2}$	$\boldsymbol{\beta_{eq}}$	qual.	low	0	12	6.3	7.6	12.0	12
12	$\boldsymbol{\Sigma_3}$	$\boldsymbol{\beta_{eq}}$	qual.	low	0	12	6.8	7.9	10.0	12
12	$\boldsymbol{\Sigma_1}$	$\boldsymbol{\beta_v}$	no	low	0	8	6.9	6.8	4.8	12
12	$\boldsymbol{\Sigma_2}$	$\boldsymbol{\beta_v}$	no	low	0	8	6.1	6.7	12.0	12
12	$\boldsymbol{\Sigma_3}$	$\boldsymbol{\beta_v}$	no	low	0	8	6.3	7.0	9.5	12
12	$\boldsymbol{\Sigma_1}$	$\boldsymbol{\beta_v}$	quant.	low	0	8	6.8	6.7	4.7	12
12	$\boldsymbol{\Sigma_2}$	$\boldsymbol{\beta_v}$	quant.	low	0	8	6.0	6.8	12.0	12
12	$\boldsymbol{\Sigma_3}$	$\boldsymbol{\beta_v}$	quant.	low	0	8	6.4	7.2	9.5	12
12	$\boldsymbol{\Sigma_1}$	$\boldsymbol{\beta_v}$	qual.	low	0	8	6.9	6.6	4.5	12
12	$\boldsymbol{\Sigma_2}$	$\boldsymbol{\beta_v}$	qual.	low	0	8	6.0	6.9	12.0	12
12	$\boldsymbol{\Sigma_3}$	$\boldsymbol{\beta_v}$	qual.	low	0	8	6.4	7.3	9.5	12
12	$\boldsymbol{\Sigma_1}$	$\boldsymbol{\beta_{eq}}$	no	high	0	12	5.3	5.2	2.8	12
12	$\boldsymbol{\Sigma_2}$	$\boldsymbol{\beta_{eq}}$	no	high	0	12	5.0	5.8	12.0	12
12	$\boldsymbol{\Sigma_3}$	$\boldsymbol{\beta_{eq}}$	no	high	0	12	5.1	5.8	9.3	12
12	$\boldsymbol{\Sigma_1}$	$\boldsymbol{\beta_{eq}}$	quant.	high	0	12	5.3	5.1	2.7	12
12	$\boldsymbol{\Sigma_2}$	$\boldsymbol{\beta_{eq}}$	quant.	high	0	12	5.1	6.0	12.0	12
12	$\boldsymbol{\Sigma_3}$	$\boldsymbol{\beta_{eq}}$	quant.	high	0	12	5.1	6.0	9.3	12
12	$\boldsymbol{\Sigma_1}$	$\boldsymbol{\beta_{eq}}$	qual.	high	0	12	5.2	5.0	2.6	12
12	$\boldsymbol{\Sigma_2}$	$\boldsymbol{\beta_{eq}}$	qual.	high	0	12	5.0	6.1	12.0	12
12	$\boldsymbol{\Sigma_3}$	$\boldsymbol{\beta_{eq}}$	qual.	high	0	12	5.1	6.1	9.3	12
12	$\boldsymbol{\Sigma_1}$	$\boldsymbol{\beta_v}$	no	high	0	8	5.7	5.7	3.6	12
12	$\boldsymbol{\Sigma_2}$	$\boldsymbol{\beta_v}$	no	high	0	8	4.9	5.5	12.0	12
12	$\boldsymbol{\Sigma_3}$	$\boldsymbol{\beta_v}$	no	high	0	8	5.2	5.8	9.1	12
12	$\boldsymbol{\Sigma_1}$	$\boldsymbol{\beta_v}$	quant.	high	0	8	5.7	5.7	3.5	12
12	$\boldsymbol{\Sigma_2}$	$\boldsymbol{\beta_v}$	quant.	high	0	8	5.0	5.7	12.0	12
12	$\boldsymbol{\Sigma_3}$	$\boldsymbol{\beta_v}$	quant.	high	0	8	5.2	5.9	9.1	12
12	$\boldsymbol{\Sigma_1}$	$\boldsymbol{\beta_v}$	qual.	high	0	8	5.8	5.6	3.5	12
12	$\boldsymbol{\Sigma_2}$	$\boldsymbol{\beta_v}$	qual.	high	0	8	4.9	5.8	12.0	12
12	$\boldsymbol{\Sigma_3}$	$\boldsymbol{\beta_v}$	qual.	high	0	8	5.4	6.2	9.2	12

Table S.1: Mean number of additionally included prognostic variables for all scenarios with $K = 12$.
