# Supplementary material for: A simulation study on estimating biomarker–treatment interaction effects in randomized trials with prognostic variables
Source: Trials. 2018 Feb 20;19:128. doi: 10.1186/s13063-018-2491-0 (PMC5819679; doi:10.1186/s13063-018-2491-0)
Supplement: Supplementary file 8 — Table S2. Mean number of additionally included prognostic variables for all scenarios with K=24. (PDF 68 kb) [file 13063_2018_2491_MOESM8_ESM.pdf]

K	$\boldsymbol{\Sigma}$	$\boldsymbol{\beta_k}$	**Interact.**	**Cens.**	**Main**	**True**	**AIC$_\mathbf{A}$**	**AIC$_\mathbf{B}$**	**Sig**	**Full**
24	$\boldsymbol{\Sigma_1}$	$\boldsymbol{\beta_{eq}}$	no	low	0	24	14.0	13.6	7.4	24
24	$\boldsymbol{\Sigma_2}$	$\boldsymbol{\beta_{eq}}$	no	low	0	24	12.7	13.6	24.0	24
24	$\boldsymbol{\Sigma_3}$	$\boldsymbol{\beta_{eq}}$	no	low	0	24	13.2	14.1	21.2	24
24	$\boldsymbol{\Sigma_1}$	$\boldsymbol{\beta_{eq}}$	quant.	low	0	24	14.0	13.4	7.3	24
24	$\boldsymbol{\Sigma_2}$	$\boldsymbol{\beta_{eq}}$	quant.	low	0	24	12.6	13.6	24.0	24
24	$\boldsymbol{\Sigma_3}$	$\boldsymbol{\beta_{eq}}$	quant.	low	0	24	13.2	14.2	21.1	24
24	$\boldsymbol{\Sigma_1}$	$\boldsymbol{\beta_{eq}}$	qual.	low	0	24	13.8	12.9	7.0	24
24	$\boldsymbol{\Sigma_2}$	$\boldsymbol{\beta_{eq}}$	qual.	low	0	24	12.5	13.7	24.0	24
24	$\boldsymbol{\Sigma_3}$	$\boldsymbol{\beta_{eq}}$	qual.	low	0	24	13.2	14.2	21.1	24
24	$\boldsymbol{\Sigma_1}$	$\boldsymbol{\beta_v}$	no	low	0	16	13.7	13.4	8.8	24
24	$\boldsymbol{\Sigma_2}$	$\boldsymbol{\beta_v}$	no	low	0	16	12.0	12.6	24.0	24
24	$\boldsymbol{\Sigma_3}$	$\boldsymbol{\beta_v}$	no	low	0	16	12.5	13.2	20.6	24
24	$\boldsymbol{\Sigma_1}$	$\boldsymbol{\beta_v}$	quant.	low	0	16	13.6	13.4	8.8	24
24	$\boldsymbol{\Sigma_2}$	$\boldsymbol{\beta_v}$	quant.	low	0	16	12.0	12.7	24.0	24
24	$\boldsymbol{\Sigma_3}$	$\boldsymbol{\beta_v}$	quant.	low	0	16	12.5	13.3	20.6	24
24	$\boldsymbol{\Sigma_1}$	$\boldsymbol{\beta_v}$	qual.	low	0	16	13.6	13.2	8.4	24
24	$\boldsymbol{\Sigma_2}$	$\boldsymbol{\beta_v}$	qual.	low	0	16	12.0	12.8	24.0	24
24	$\boldsymbol{\Sigma_3}$	$\boldsymbol{\beta_v}$	qual.	low	0	16	12.5	13.5	20.6	24
24	$\boldsymbol{\Sigma_1}$	$\boldsymbol{\beta_{eq}}$	no	high	0	24	10.5	10.3	5.2	24
24	$\boldsymbol{\Sigma_2}$	$\boldsymbol{\beta_{eq}}$	no	high	0	24	10.0	10.8	24.0	24
24	$\boldsymbol{\Sigma_3}$	$\boldsymbol{\beta_{eq}}$	no	high	0	24	10.2	10.9	20.2	24
24	$\boldsymbol{\Sigma_1}$	$\boldsymbol{\beta_{eq}}$	quant.	high	0	24	10.4	10.1	5.2	24
24	$\boldsymbol{\Sigma_2}$	$\boldsymbol{\beta_{eq}}$	quant.	high	0	24	10.1	11.0	24.0	24
24	$\boldsymbol{\Sigma_3}$	$\boldsymbol{\beta_{eq}}$	quant.	high	0	24	10.2	11.1	20.3	24
24	$\boldsymbol{\Sigma_1}$	$\boldsymbol{\beta_{eq}}$	qual.	high	0	24	10.4	9.9	4.9	24
24	$\boldsymbol{\Sigma_2}$	$\boldsymbol{\beta_{eq}}$	qual.	high	0	24	10.1	11.1	24.0	24
24	$\boldsymbol{\Sigma_3}$	$\boldsymbol{\beta_{eq}}$	qual.	high	0	24	10.2	11.2	20.3	24
24	$\boldsymbol{\Sigma_1}$	$\boldsymbol{\beta_v}$	no	high	0	16	11.5	11.2	6.8	24
24	$\boldsymbol{\Sigma_2}$	$\boldsymbol{\beta_v}$	no	high	0	16	9.9	10.6	24.0	24
24	$\boldsymbol{\Sigma_3}$	$\boldsymbol{\beta_v}$	no	high	0	16	10.3	10.9	19.7	24
24	$\boldsymbol{\Sigma_1}$	$\boldsymbol{\beta_v}$	quant.	high	0	16	11.4	11.2	6.7	24
24	$\boldsymbol{\Sigma_2}$	$\boldsymbol{\beta_v}$	quant.	high	0	16	9.9	10.7	24.0	24
24	$\boldsymbol{\Sigma_3}$	$\boldsymbol{\beta_v}$	quant.	high	0	16	10.4	11.1	19.7	24
24	$\boldsymbol{\Sigma_1}$	$\boldsymbol{\beta_v}$	qual.	high	0	16	11.4	11.2	6.6	24
24	$\boldsymbol{\Sigma_2}$	$\boldsymbol{\beta_v}$	qual.	high	0	16	10.0	10.9	24.0	24
24	$\boldsymbol{\Sigma_3}$	$\boldsymbol{\beta_v}$	qual.	high	0	16	10.7	11.5	19.8	24

Table S.2: Mean number of additionally included prognostic variables for all scenarios with $K = 24$.
