# Supplementary material for: A simulation study on estimating biomarker–treatment interaction effects in randomized trials with prognostic variables
Source: Trials. 2018 Feb 20;19:128. doi: 10.1186/s13063-018-2491-0 (PMC5819679; doi:10.1186/s13063-018-2491-0)
Supplement: Supplementary file 9 — Table S3. Mean number of additionally included prognostic variables for all scenarios with K=36. (PDF 68 kb) [file 13063_2018_2491_MOESM9_ESM.pdf]

K	$\boldsymbol{\Sigma}$	$\boldsymbol{\beta_k}$	**Interact.**	**Cens.**	**Main**	**True**	$\mathbf{AIC_A}$	$\mathbf{AIC_B}$	**Sig**	**Full**
36	$\boldsymbol{\Sigma_1}$	$\boldsymbol{\beta_{eq}}$	no	low	0	36	20.6	20.1	10.6	36
36	$\boldsymbol{\Sigma_2}$	$\boldsymbol{\beta_{eq}}$	no	low	0	36	18.2	19.2	36.0	36
36	$\boldsymbol{\Sigma_3}$	$\boldsymbol{\beta_{eq}}$	no	low	0	36	20.0	20.9	27.2	36
36	$\boldsymbol{\Sigma_1}$	$\boldsymbol{\beta_{eq}}$	quant.	low	0	36	20.5	19.8	10.3	36
36	$\boldsymbol{\Sigma_2}$	$\boldsymbol{\beta_{eq}}$	quant.	low	0	36	18.3	19.2	36.0	36
36	$\boldsymbol{\Sigma_3}$	$\boldsymbol{\beta_{eq}}$	quant.	low	0	36	19.9	20.9	27.1	36
36	$\boldsymbol{\Sigma_1}$	$\boldsymbol{\beta_{eq}}$	qual.	low	0	36	20.3	19.0	9.9	36
36	$\boldsymbol{\Sigma_2}$	$\boldsymbol{\beta_{eq}}$	qual.	low	0	36	18.2	19.2	36.0	36
36	$\boldsymbol{\Sigma_3}$	$\boldsymbol{\beta_{eq}}$	qual.	low	0	36	19.9	20.8	27.1	36
36	$\boldsymbol{\Sigma_1}$	$\boldsymbol{\beta_v}$	no	low	0	24	20.4	20.1	12.5	36
36	$\boldsymbol{\Sigma_2}$	$\boldsymbol{\beta_v}$	no	low	0	24	17.6	18.3	36.0	36
36	$\boldsymbol{\Sigma_3}$	$\boldsymbol{\beta_v}$	no	low	0	24	19.2	19.8	27.4	36
36	$\boldsymbol{\Sigma_1}$	$\boldsymbol{\beta_v}$	quant.	low	0	24	20.5	20.0	12.4	36
36	$\boldsymbol{\Sigma_2}$	$\boldsymbol{\beta_v}$	quant.	low	0	24	17.6	18.3	36.0	36
36	$\boldsymbol{\Sigma_3}$	$\boldsymbol{\beta_v}$	quant.	low	0	24	19.1	19.9	27.3	36
36	$\boldsymbol{\Sigma_1}$	$\boldsymbol{\beta_v}$	qual.	low	0	24	20.4	19.6	11.9	36
36	$\boldsymbol{\Sigma_2}$	$\boldsymbol{\beta_v}$	qual.	low	0	24	17.6	18.3	36.0	36
36	$\boldsymbol{\Sigma_3}$	$\boldsymbol{\beta_v}$	qual.	low	0	24	19.0	19.9	27.2	36
36	$\boldsymbol{\Sigma_1}$	$\boldsymbol{\beta_{eq}}$	no	high	0	36	15.6	15.4	7.4	36
36	$\boldsymbol{\Sigma_2}$	$\boldsymbol{\beta_{eq}}$	no	high	0	36	15.2	16.0	36.0	36
36	$\boldsymbol{\Sigma_3}$	$\boldsymbol{\beta_{eq}}$	no	high	0	36	14.9	15.6	26.0	36
36	$\boldsymbol{\Sigma_1}$	$\boldsymbol{\beta_{eq}}$	quant.	high	0	36	15.7	15.4	7.5	36
36	$\boldsymbol{\Sigma_2}$	$\boldsymbol{\beta_{eq}}$	quant.	high	0	36	15.1	16.1	36.0	36
36	$\boldsymbol{\Sigma_3}$	$\boldsymbol{\beta_{eq}}$	quant.	high	0	36	14.8	15.7	26.1	36
36	$\boldsymbol{\Sigma_1}$	$\boldsymbol{\beta_{eq}}$	qual.	high	0	36	15.5	14.9	7.1	36
36	$\boldsymbol{\Sigma_2}$	$\boldsymbol{\beta_{eq}}$	qual.	high	0	36	15.2	16.1	36.0	36
36	$\boldsymbol{\Sigma_3}$	$\boldsymbol{\beta_{eq}}$	qual.	high	0	36	15.0	16.0	26.1	36
36	$\boldsymbol{\Sigma_1}$	$\boldsymbol{\beta_v}$	no	high	0	24	17.3	17.1	9.6	36
36	$\boldsymbol{\Sigma_2}$	$\boldsymbol{\beta_v}$	no	high	0	24	15.0	15.6	36.0	36
36	$\boldsymbol{\Sigma_3}$	$\boldsymbol{\beta_v}$	no	high	0	24	15.5	16.1	26.2	36
36	$\boldsymbol{\Sigma_1}$	$\boldsymbol{\beta_v}$	quant.	high	0	24	17.2	16.9	9.5	36
36	$\boldsymbol{\Sigma_2}$	$\boldsymbol{\beta_v}$	quant.	high	0	24	15.0	15.8	36.0	36
36	$\boldsymbol{\Sigma_3}$	$\boldsymbol{\beta_v}$	quant.	high	0	24	15.6	16.3	26.2	36
36	$\boldsymbol{\Sigma_1}$	$\boldsymbol{\beta_v}$	qual.	high	0	24	17.1	16.6	9.5	36
36	$\boldsymbol{\Sigma_2}$	$\boldsymbol{\beta_v}$	qual.	high	0	24	15.1	15.9	36.0	36
36	$\boldsymbol{\Sigma_3}$	$\boldsymbol{\beta_v}$	qual.	high	0	24	16.3	17.1	26.5	36

Table S.3: Mean number of additionally included prognostic variables for all scenarios with $K = 36$.
